# Supplementary material for: Searching the druggable genome using large language models
Source: Bioinformatics. 2026 Aug 22;42(9):btag632. doi: 10.1093/bioinformatics/btag632 (PMC13544961; doi:10.1093/bioinformatics/btag632)
Supplement: Supplementary file 1 [file btag632_supplementary_data.docx]

**SUPPLEMENTAL**

**Implementation and Reproducibility Details**

An archived snapshot of the evaluation repository, including the evaluation scripts, the exact sampled input sets, and run_experiments.sh, is permanently available on Zenodo (DOI: 10.5281/zenodo.20938813). The actively maintained version is hosted on GitHub at <https://github.com/griffithlab/dgidb-mcp-server>. All GPT-5 evaluations were conducted using model version gpt-5-2025-08-07, corresponding to the first available release. GPT-5 does not expose temperature as a configurable parameter; evaluations were therefore run at the default temperature (T=1), which limits strict reproducibility. Results should be interpreted as representative of typical model behavior rather than as fully deterministic outputs.

Agent orchestration used the OpenAI Agents SDK for Python with the OpenAI client. MCP tools were defined with FastMCP and launched as a subprocess via the fastmcp CLI over the stdio transport, under Python 3.11.7. The complete benchmark is driven by run_experiments.sh, an entry-point script in the project repository, which installs the pinned dependencies from requirements.txt and runs all evaluation tasks with a single command (bash GPT_eval/src/run_experiments.sh). Tool use was not enforced: ModelSettings did not override the SDK's default tool_choice="auto", so the model decided on each turn whether to invoke a tool, and Runner.run() was called without an explicit max_turns, leaving the SDK's default cap of 10 model-call/tool-execution cycles per query. Each query instantiated a fresh MCP server subprocess and a fresh agent run, with no tool-call history shared across items. The joint ranking task issued one LLM call to rank candidate genes per disease-therapy combination and one additional independent call per gene to rank candidate drugs. The GPT-5-alone baseline used the identical model version and temperature with no MCP tools attached.

The deployed system comprises two separate MCP servers (DGIdb and CIViC), and the worked example in Figure 1 was produced with Claude connected to both. For the quantitative benchmark we used a simplified local harness that exposed the same tools through a single process. This does not change what the model experiences: the Agents SDK presents the union of attached MCP tools to the model as a single flat tool list, so the tool definitions available for selection and multi-hop chaining are identical whether they originate from one combined server or two separate ones. To keep the deployment architecture distinct from the evaluation setup, we refer to the benchmarked condition as "GPT-5 + MCP tools".

All DGIdb and CIViC queries were issued against the live public APIs in January 2026. Because both are actively curated resources, retrieved annotations, and therefore exact metric values, may differ when queries are repeated at a later date.

**Table 1: DGIdb MCP Example Use Cases**

| **Category** | **Example Query** | **Intended Output*** |
| --- | --- | --- |
| Drug Discovery | Which FDA-approved drugs target the gene KIT? | A ranked list of FDA-approved KIT-interacting drugs with interaction scores, interaction types, approval status, and links to supporting sources. [Chat Link](https://claude.ai/share/62d8e788-7b0f-453a-af08-dc066d34b9b1). |
| Drug Info | What information is available about Imatinib? | A structured overview of Imatinib including its FDA approval status, year of approval, therapeutic and drug class annotations. [Chat Link](https://claude.ai/share/999dfa2e-aa4b-4a5c-bcf7-9be72886b873). |
| Gene Category Info | What categories and functional annotations are associated with the gene BRAF? | A structured summary of BRAF annotations, including clinical actionability, drug resistance relevance, druggability, enzyme class, and kinase classification, with the supporting source databases listed for each category. [Chat Link](https://claude.ai/share/91a62f7d-d278-4a4b-a70c-8f54d598d147). |
| Gene Symbol Disambiguation | Is FLT3 an unambiguous gene symbol? | An assessment of whether FLT3 maps uniquely to a single gene, including any known aliases/synonyms, overlapping symbols, and the resolved canonical gene identifier(s), with supporting source links. [Chat Link](https://claude.ai/share/5fa0883b-538b-47c8-8b68-99b8d9db4045). |
| Mechanism-specific targeting | Which inhibitors interact with EGFR? | A filtered list of EGFR inhibitors, prioritized by FDA approval and interaction score, with evidence sources cited. [Chat Link](https://claude.ai/share/0daf9c70-b6cd-4c00-b2af-ff2c91a6aff7). |
| Interaction type interpretation | What genes interact with the drug Ibrutinib and by what mechanism of interaction? | A structured summary of Ibrutinib-gene interactions, annotated with interaction direction and type (e.g., inhibitor, modulator). [Chat Link](https://claude.ai/share/1297b4d8-d55b-408d-9648-ef34e266f7ca). |

Chat links to conversations with Opus 4.5 via Claude Desktop on 1/14/2026.

*API responses are not visible in the linked chats.

**Table 2: DGIdb MCP + CIViC MCP Use Cases.**

| **Category** | **Example Query** | **Intended Output**^1^ |
| --- | --- | --- |
| Resistance-guided drug discovery^2^  (CIViC→DGIdb) | What genes can cause resistance to ibrutinib in chronic lymphocytic leukemia, and what alternative drugs can target them? | CIViC-supported resistance genes for ibrutinib in CLL and a ranked list of interacting alternative drugs from DGIdb, with citations. [Chat Link](https://claude.ai/share/be18a9b9-0a1b-4831-9dc9-9f72dd9d5049). |
| Oncogenicity-driven functional validation  (CIViC→DGIdb) | What is the evidence for oncogenicity of variants in ERBB2 for breast cancer, and what inhibitors could I use to experimentally validate their importance in vitro? | CIViC-supported oncogenic ERBB2 variants in breast cancer with summarized evidence and citations, plus a ranked list of ERBB2-targeting inhibitors from DGIdb suitable for in vitro validation. [Chat Link](https://claude.ai/share/1bd81002-fe12-44aa-9b5c-775a0fb426d2). |
| Drug target profiling with clinical evidence  (DGIdb→CIViC) | What genes interact with the drug Ibrutinib, and which of those genes have specific variants in CIViC associated with sensitivity or resistance? | A list of genes interacting with ibrutinib annotated with CIViC sensitivity or resistance evidence where available. [Chat Link](https://claude.ai/share/9ad52a71-74c1-40a5-aa22-afc03e5fa940). |
| Target resistance interpretation (DGIdb→CIViC) | Are there known resistance mechanisms for genes targeted by Osimertinib? | Osimertinib targets genes with CIViC-documented resistance mechanisms and supporting evidence links. [Chat Link](https://claude.ai/share/9da56313-74b4-4978-954b-43437a63e47f). |
| Gene-guided therapy matching  (DGIdb→CIViC) | Which drugs interact with PIK3CA, and is there evidence of sensitivity to these drugs in breast cancer? | PIK3CA-interacting drugs prioritized by DGIdb and annotated with CIViC sensitivity evidence in breast cancer. [Chat Link](https://claude.ai/share/b73748ee-74d6-4b7f-92f1-8a8620960bcd). |
| Expression-based therapy matching  (DGIdb→CIViC) | For tumors with EGFR amplification, which drugs target EGFR and is there clinical evidence supporting sensitivity? | EGFR-targeting drugs with CIViC evidence supporting sensitivity in EGFR-amplified tumors. [Chat Link](https://claude.ai/share/b2d6ffa7-a2a2-4c2f-9805-3d6aad577362). |

Chat links to conversations with Opus 4.5 via Claude Desktop on 1/14/2026 and 3/25/2026.

**^1^** API responses are not visible in the linked chats.

**^2^** Illustrated step-by-step in Figure 1. The query shown here is a simplified natural-language paraphrase of the use case; the actual prompt used to produce the Figure 1 run (shown in the figure) named both CIViC and DGIdb explicitly to reliably elicit the depicted tool-calling sequence.

**Table 3.** **Drug classification performance.** N represents the number of positive samples for each label.

|  | **GPT-5 + DGIdb MCP** | | | **GPT-5** | | |
| --- | --- | --- | --- | --- | --- | --- |
| **Label** | **Precision** | **Recall** | **F1** | **Precision** | **Recall** | **F1** |
| FDA-Approved  (N=48) | 1.00 | 0.98 | 0.99 | 0.89 | 1.00 | 0.94 |
| Immunotherapy (N=47) | 1.00 | 1.00 | 1.00 | 0.95 | 0.38 | 0.55 |
| Antineoplastic  (N=64) | 1.00 | 1.00 | 1.00 | 0.86 | 0.69 | 0.77 |

**Table 4. Drug classification performance.** GPT-5 with access to the DGIdb MCP server but with no mention of DGIdb in the prompt. Here, N represents how samples the MCP server was used for when the associated label was positive.

|  | **MCP Used (N=45)** | | | | **No MCP Usage (N=55)** | | | |
| --- | --- | --- | --- | --- | --- | --- | --- | --- |
| **Label** | **N** | **Precision** | **Recall** | **F1** | **N** | **Precision** | **Recall** | **F1** |
| FDA-Approved | 10/45 | 0.91 | 1.00 | 0.95 | 38/55 | 0.93 | 1.00 | 0.96 |
| Immunotherapy | 15/45 | 1.00 | 1.00 | 1.00 | 32/55 | 0.95 | 0.68 | 0.80 |
| Antineoplastic | 17/45 | 1.00 | 0.88 | 0.94 | 47/55 | 0.91 | 0.68 | 0.78 |

**Randomly sampled examples:**

**No MCP Usage:** TAMOXIFEN, TREMELIMUMAB, RITUXIMAB, NIVOLUMAB, PEGINTERFERON ALFA-2A, [125I]TYR45-KISSPEPTIN-15, [D-TYR8]CYN 154806, DULANERMIN, ENTRECTINIB, GOLIMUMAB

**MCP used:** ODM-203, LBY-135, ISOBAVACHALCONE, INTERFERON ALFA-N3, CHEMBL_CHEMBL19954, CIXUTUMUMAB, ABT-239, (+)-ADTN, CS+, JNK INHIBITOR 9L

**Table 5. Drug Candidate Identification Performance.** Duplicate genes are not counted multiple times towards the DGIdb Drug List results.

| **Model** | **Metric** | **CIViC Gene List** | **DGIdb Drug List** |
| --- | --- | --- | --- |
| **GPT-5 + MCP Tools** | Precision | 0.96 | 0.95 |
|  | Recall | 0.96 | 0.97 |
|  | F1-score | 0.96 | 0.96 |
|  | NDCG | 0.97 | 0.94 |
| **GPT-5** | Precision | 0.23 | 0.17 |
|  | Recall | 0.43 | 0.19 |
|  | F1-score | 0.30 | 0.18 |
|  | NDCG | 0.44 | 0.25 |

**Prompt 1: DGIdb Drug Info**

**System:** "You are a chatbot for the Drug Gene Interaction Database (DGIdb)"

**User:** “Drug: {drug_name}

For the drug named above ONLY, state if each property applies (YES/NO) based on DGIdb.

Output EXACTLY this 2-line CSV (no extra text):

fda_approval,immunotherapy,anti_neoplastic

<YES/NO>,<YES/NO>,<YES/NO>”

**Prompt 2: DGIdb Drug Info w/ MCP, no mention of DGIdb**

**System:** “You are a helpful AI assistant with access to external tools. Follow the user's instructions carefully.”

**User:** “Drug: {drug_name}

For the drug named above ONLY, state if each property applies (YES/NO).

Output EXACTLY this 2-line CSV (no extra text):

fda_approval,immunotherapy,anti_neoplastic

<YES/NO>,<YES/NO>,<YES/NO>”

**Prompt 3: CIViC Genes Responsible For Resistance**

**System:** "You are a chatbot for CIViC and the Drug Gene Interaction Database (DGIdb). "

When listing genes from CIViC, you must rank them using CIViC evidence strength."

GENE RANKING RULES:

1. Rank genes first by evidenceLevel (A strongest → E weakest).

2. Within the same evidenceLevel, rank genes by evidenceRating (5 → 1 → null).

3. Return only HGNC gene symbols, in this ranked order, as a comma-separated list

like: GENE1,GENE2,GENE3."

**User: “**According to CIViC, list genes whose variants/molecular profiles are associated with resistance to {therapies} in {disease_name} (significance = RESISTANCE)”

**Prompt 4: DGIdb Candidate Drugs**

**System:** “You are a chatbot for the CIViC and Drug Gene Interaction Database (DGIdb). You receive a gene and DGIdb interaction for that gene. Your task is to return a ranked list of up to 20 drugs.

RANKING RULES:

1. List FDA-approved drugs first.

2. Within the approved drugs, sort by the DGIdb interactionScore in descending order

3. After all approved drugs, list non-approved drugs, also sorted by interactionScore in descending order.

4. Return only drug names (no scores), as a comma-separated list like: DRUG1,DRUG2.”

**User:** “What drugs target or modulate this gene product in DGIdb? Gene: {gene}

Return a comma-separated list of up to 20 drug names following the ranking rules.”

**Suppl. Figure 1.** **Evaluation workflow overview.** The LLM is first prompted to provide the list of ordered genes. Then individually prompted to provide the list of interacting drugs for each gene.
